# Insights on Bmi-1 therapeutic targeting in head and neck cancers

**DOI:** 10.32604/or.2024.053764

**Published:** 2025-01-16

**Authors:** JESSIE REYES-CARMONA

**Affiliations:** LICIFO, Department of Restorative Sciences, Faculty of Dentistry, University of Costa Rica (HNSCC), San José, 11501, Costa Rica

**Keywords:** Cancer stem cell (CSC), B lymphoma Mo-MLV insertion region 1 homolog Bmi-1, Therapeutic resistance, Head and neck squamous cell carcinoma (HNSCC), Targeted therapy

## Abstract

The B lymphoma Mo-MLV insertion region 1 homolog (Bmi-1) protein of the polycomb complex is an essential mediator of the epigenetic transcriptional silencing by the chromatin structure. It has been reported to be crucial for homeostasis of the stem cells and tumorigenesis. Though years of investigation have clarified Bmi-1’s transcriptional regulation, post-translational modifications, and functions in controlling cellular bioenergetics, pathologies, and DNA damage response, the full potential of this protein with so many diverse roles are still unfulfilled. Bmi-1 is overexpressed in many human malignancies. Unraveling the Bmi-1’s precise functional role in head and neck cancers can be attractive for mechanisms-based developmental therapeutics. This review attempts to synthesize the current knowledge on Bmi-1 with an emphasis on the role that Bmi-1 plays in oral cancer progression and evaluates how this can be used in advancing clinical treatment strategies for head and neck cancer. Bmi-1 is a promising target for therapy because it has been linked to a stemness and oncogenesis signature. However, to use Bmi-1 as a prognostic marker and a therapeutic target in the long run, new methods are imperative for further characterization of the physiological roles of Bmi-1. Current biological insights of Bmi-1 as a master regulator of stem cell self-renewal have emerged as a prominent player in cancer stem cell (CSC) biology. Bmi-1+ cells mediate chemoresistance and metastasis. On the other hand, inhibiting Bmi-1 rescinds CSC function and re-sensitizes cancer cells to chemotherapy. Therefore, elucidating therapeutic approaches targeting Bmi-1 can be leveraged to further research analysis to advance clinical treatment strategies for head and neck cancer.

## Introduction

Tumors that arise in the salivary glands, nose, pharynx, mouth cavity, and other parts of the head and neck region are commonly referred to as head and neck cancers. About 400,000 deaths and more than 900,000 new diagnoses of head and neck squamous cell carcinoma (HNSCC) are reported each year, making it the sixth most prevalent cancer type in terms of incidence [1]. The Third National Cancer Survey found a five-year cause-specific survival rate of 56% for patients with HNSCC, with variations based on sex and race [2]. According to current trends, approximately 40,000 new cases of HNSCC are expected to be diagnosed in the US every year [3,4]. To improve patient outcomes and develop targeted therapies, comprehending the molecular mechanisms underlying the development and progression of HNSCC is imperative.

Since cancer stem cells (CSCs) are involved in all of these extremely aggressive cancers, treatment resistance is triggered, which frequently results in relapse [5]. Bmi-1, a protein that is a member of the polycomb group, has been regarded as a promising target for therapeutic intervention. This protein is essential for several cellular processes, including cell cycle regulation, differentiation, senescence, and renewal [6]. This review focuses on Bmi-1’s involvement in head and neck cancer, a condition for which it is assumed to be a promising target for implementing innovative therapeutic interventions.

## Biomarkers in Head and Neck Cancer

Several biomarkers, both molecular and clinical, are important in the diagnosis, prognosis, and management of cancer. Markers such as Epidermal Growth Factor Receptor (EGFR), p16 status, tumor Protein p53, Bmi-1, and Programmed Death-Ligand 1 (PD-L1) expression often take precedence due to their direct implications for treatment decisions. Biomarkers play crucial roles in understanding the biology of cancer, guiding treatment decisions, predicting patient outcomes, and developing targeted therapies. Ongoing research continues to identify new markers and define their clinical significance in the management of head and neck cancer.

## Bmi-1 Protein

**Figure 1:**
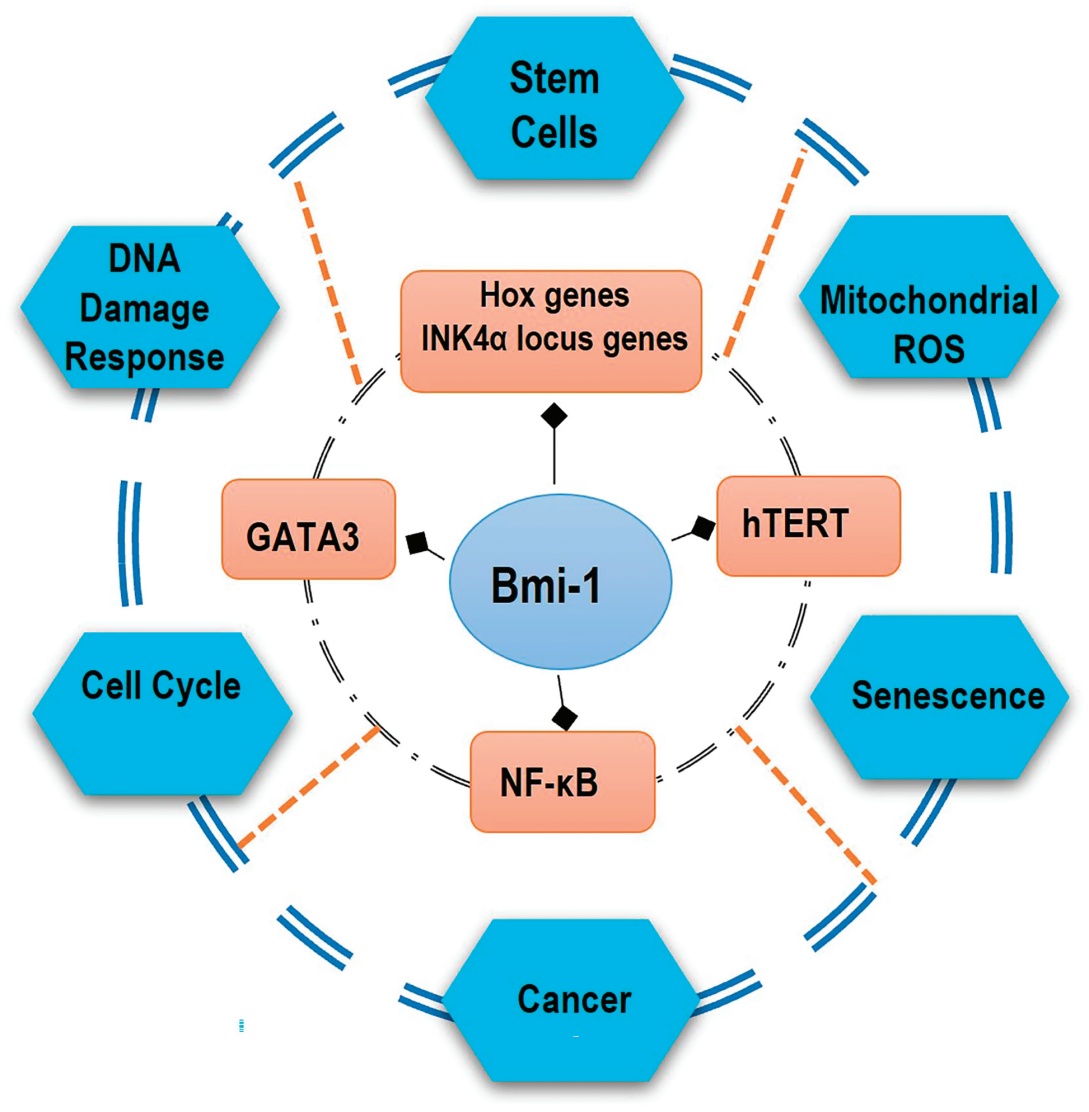
Bmi-1 can activate various molecular pathways, participating in important biological functions of the body. Bmi-1 can regulate stem cell self-renewal through the suppression of Hox genes and the INK4a locus genes p16^Ink4a^ and p19^Arf^ and, also several cellular mechanisms by the activation of the telomerase, transcriptional factor GATA3, and NF-κB pathway. Some of the genes involved in stem-cell-fate decisions are likely to include the prevention from senescence, apoptosis, DNA response damage, ROS, and differentiation but rather induction of immortalization and promotion of proliferation of cancer.

The Bmi-1 gene is localized on chromosome 10 (10p11.23) and encodes a 37 kDa protein comprising 326 amino acids [1]. Bmi-1 is implied in several biological processes such as embryonic development, organ formation, tumorigenesis, stabilization, and differentiation of stem cells [1,7,8]. Moreover, Bmi-1 is expressed in almost all tissues given that it is involved in various cellular processes such as DNA damage response, senescence, cell cycle, self-renewal of stem cells, mitochondrial reactive oxygen species, and metabolism of cancer cells (Fig. 1).

## Bmi-1 Mechanisms in Head and Neck Cancers

Bmi-1 expression is frequently upregulated in various types of human cancers. Table 1 summarizes Bmi-1’s recognized roles in head and neck cancers. In this context, tumor-causing cells express Bmi-1 at a higher level than do healthy cells. Moreover, overexpression of Bmi-1 has been linked to drug resistance and tumor growth, making it a promising therapeutic target in recent years [1].

**Table 1:** Bmi-1’s recognized roles in head and neck cancer

Cancer localization	Bmi-1 role	Reference
Head and neck	Direct regulation of the EMT regulator, Twist1.	[9]
Lymphoma	Accelerate the onset of pre-B and B lymphomas, promoting lymphomagenesis directly within T- and B-lymphocyte lineages and in collaboration with the myc gene in tumorigenesis.	[10]
Naso-pharyngeal	Lead to the immortalization of nasopharyngeal epithelial cells, the induction of telomerase activity, and the decrease of p16^Ink4a^ expression.	[11–13]
Tongue	Atypical overexpression of Bmi-1 is linked to cervical node metastases.	[14]
Salivary Glands	In Mucoepidermoid carcinoma (MEC) p53 activation does not induce MEC CSC apoptosis, it reduces stemness properties such as self-renewal by regulating Bmi-1 expression. Therapeutic activation of p53 by silencing Bmi-1, prevented CSC-mediated tumor recurrence in preclinical trials.	[15]
Salivary gland carcinosarcoma	In Salivary adenoid cystic carcinoma (AdCC) Metastasis is linked to overexpression of Bmi-1. It plays a crucial role in AdCC progression by interaction with EMT-related markers and predict poor survival.	[16]
	In adenoid cystic carcinoma (ACC) Bmi-1 overexpression was correlated with high proliferative rate and unfavorable outcome.	

Over the years, the relevance of CSCs in the initiation and progression of diverse malignancies has gained increased recognition [6]. Since head and neck CSCs are the primary source of tumorigenesis, Bmi-1 is highly expressed in these cells [1,8]. In HNSCC, silencing Bmi-1 reduces stemness and tumor formation. Moreover, Bmi-1 is responsible for regulating stem cell self-renewal, playing a crucial role in preserving stem cell populations [5]. This is accomplished by inhibiting the INK4a-ARF locus, which encodes tumor suppressor proteins p16^Ink4a^ and p19^Arf^ [1], retaining stem cells from aging and dying [9]. Tumor formation occurs *in vivo* when p16^Ink4a^ and p19^Arf^ expression are decreased due to upregulation of Bmi-1 [10].

## The Role of p53 in Bmi-1 Expression

Often referred to as the “guardian of the genome,” the p53 tumor suppressor plays a critical role in apoptosis and serves to stop abnormal cells from proliferating and causing cancer [11]. Bmi-1 repression directly targets the transcriptional factor p53 [11]. Moreover, p53 and p16^Ink4a^ are both activated during senescence, and the effective inhibition of oncogene-induced hyperproliferation is facilitated by the activation of these two mechanisms [10]. Apoptosis is triggered by p53 in cells that have sustained irreversible damage from DNA, and this induction and sustenance require strict regulation. This has broad implications for therapy since, naturally, p16 and p53 are frequently the targets of inactivation in a diverse range of cancers [9]. For instance, in an *in vivo* xenograft study, HNSCC cell lines were transplanted into the tongue of immunodeficient mice [12]. After receiving a small molecule inhibitor, the tumor’s volume was halved, providing strong evidence of the potential therapeutic benefit of Bmi-1 inhibitors in treating cancer.

## Bmi-1 in Tumorigenesis and Metastasis

The function of Bmi-1 in head and neck cancers to promote metastasis has been associated with the activity in regulating the epithelial-mesenchymal transition (EMT) process [13]. Bmi-1 has been reported to provoke the expression of EMT-associated transcription factors, provoking invasive and migratory properties in CSCs [14]. These lead to an important implication for the spread of HNSCC to distant sites, contributing to the difficulty in treating advanced stages of the disease.

In addition to the direct effects on the tumor cells, Bmi-1 influences the tumor microenvironment by promoting the activation of the NF-κB and c-Jun N-terminal kinase pathways. It also modulates the secretion of several factors, such as those promoting angiogenesis, escape from immunity, and remodeling of the extracellular matrix, for the development of a permissive tumor niche for growth and dissemination [14,15]. Further, Bmi-1 has been demonstrated to support the stem cell phenotype of “adult” tissue and to be a prohibitor of normal, irreversibly established cellular lineages [16].

Possibly the best-described Bmi-1 function in cancer pathogenesis is most convincingly reported in the context of hematopoietic and central nervous system malignancies. Comparison from the same tumor sample reveals that its expression in a CSC and non-CSC population exists at higher levels in the CSC population [7].

Using cell surface markers that are also expressed by normal tissue stem cells is a common technique for differentiating CSCs from non-CSCs [1,17]. A notable enrichment of CSC activity has been discovered by classifying head and neck cancer cells according to the markers CD44 and αvβ6. After being cultured both *in vitro* and *in vivo*, these CSC populations have been found to noticeably increase Bmi-1 expression [1]. Moreover, it has been demonstrated that CSCs with upregulated Bmi-1 proliferate more than non-CSC populations and that CSC proliferation is inhibited when Bmi-1 is knocked down using shRNA [8,18].

From a clinical perspective, Bmi-1 was initially identified as an oncogene that cooperates with Myc in murine B- and T-cell lymphomas [19]. The overexpression of Bmi-1 induces stem-like characteristics linked to the induction of EMT, which in turn encourages invasion and metastasis and ultimately results in a poor prognosis. Table 1 highlights the key roles played by Bmi-1 in head and neck cancers hitherto reported. As demonstrated by a study, a considerable percentage of tongue cancers have abnormally high levels of Bmi-1 expression [14]. Moreover, cervical node metastasis is linked to elevated Bmi-1, which functions serving as an innovative biomarker in the diagnosis and prognosis of tongue cancer, as well as a major driver with multiple oncogenic functions during the disease’s progression [14,19]. However, in head and neck cancers, high expression of both Twist1 and Bmi-1 positively correlated with the expression of E-cadherin and p16Ink4a, respectively, thus drawing an association with a worse prognosis [9]. HNSCC also demonstrates substantial levels of nuclear Bmi-1 within a CD44+ fraction comprising less than 10% of the tumor, showing stem-like properties by giving rise to the tumor *in vivo* [20]. Additionally, in a study, the upregulation and overexpression of Bmi-1 were detected in a large number of tumors from patients with nasopharyngeal carcinoma, indicating a correlation between Bmi-1 and a patient’s poor prognosis and the advanced invasive phase of tumor growth [12]. Besides, Bmi-1 functions independently in nasopharyngeal carcinoma pathogenesis by partially stimulating EMT via the tumor suppressor PTEN’s target, triggering the PI3K/Akt pathway [11].

## Therapeutic Approaches: Chemotherapeutic Treatment

Drug resistance is a critical issue in cancer treatment. Cisplatin is the most frequently used chemotherapeutic drug for the treatment of HNSCC [21]. A significant proportion of patients with primary disease (30%) and relapsed patients (70%) exhibit resistance to cisplatin, indicating that intrinsic or acquired resistance restricts the effectiveness of the drug [22]. Recent evidence has unveiled that CSCs are found in perivascular niches and rely on endothelial cell-secreted factors—particularly interleukin-6 (IL-6)—for their survival and self-renewal [23]. Understanding that high IL-6 expression in serum correlates with poor survival of patients with HNSCC [24], a study evaluated the effect of cisplatin on the head and neck CSC fraction using HNSCC xenografts with humanized vasculature [24]. The authors demonstrated that cisplatin could increase the fraction of CSCs despite the lack of a significant change in the overall tumor volume. Their data also showed that cells with ALDH++ and CD44++ high expression are resistant to cisplatin treatment. Further, this study revealed that even very low concentrations of cisplatin could induce expression of the self-renewal of Bmi-1, raising the highly tumorigenic nature of these cells. This discovery partially elucidated the high rate of tumor recurrence and distant metastases seen in cisplatin-treated patients with head and neck cancers. Based on these results, a combination therapy involving a platinum-based drug and an IL-6R inhibitor might benefit patients with HNSCC [23].

In another study, the efficacy of Vorinostat, an FDA-approved histone deacetylation inhibitor (HDACi), in combined therapy with cisplatin was investigated in adenoid cystic carcinomas (ACC) [25]. The authors demonstrated that the number of CSCs in ACC cells decreased when Vorinostat was used alone. Nevertheless, after five days of Vorinostat, one of the cell lines displayed a recovery of CSCs to pre-treatment levels. These results imply that there may be a distinct “window of opportunity” for each ACC tumor when Vorinostat is administered. Combining Vorinostat and cisplatin reduces tumor viability and effectively depletes CSCs from ACC, and the ability of cisplatin to cause DNA double-strand breaks and trigger cellular senescence may be enhanced by Vorinostat, suggesting a possible mode of action [25].

## Therapeutic Approaches Targeting Bmi-1

### Small-molecule inhibitors

Several small-molecule inhibitors of Bmi-1 have been investigated across studies. The first natural product researched was Phenethyl isothiocyanate (PEITC), which is present as gluconasturtiin in cruciferous vegetables with outstanding anti-cancer effects [26]. PEITC prevents the initiation phase of the carcinogenesis process and inhibits the progression of tumorigenesis [27]. Moreover, PEITC targets multiple proteins, such as Bmi-1, to suppress various cancer-promoting mechanisms such as CSC proliferation, progression, and metastasis [26]. Furthermore, PEITC significantly decreases the expression of Bmi-1 and arrests a reduced percentage of cells in the S phase while promoting a decrease in the proportion of cells arrested in the G1 phase concomitant with the upregulation [13]. These promising results have led to the development of a series of small molecular analogs of PEITC that were designed and synthesized to discover more potent reactive oxygen species (ROS) modulators with better anticancer capacity, such as N-ethyl-4- (2-isothiocyanatoethyl) benzamide, labeled as LBL21 [28]. Notably, LBL21 demonstrates anti-proliferative ability, ROS accumulation, mitochondrial dysfunction, and CSC elimination [28].

Moreover, PTC596 (Unesbulin), a small molecule inhibitor of Bmi-1, showed satisfactory safety profiles in phase I trials [29]. For instance, an open-label, phase 1 study with multiple ascending doses was conducted to evaluate the drug’s safety and pharmacokinetics in patients with advanced solid tumors. Oral PTC596 was given every two weeks, depending on body weight, and a modified 3 + 3 scheme was used for dose escalation, with doses of 0.6, 1.3, 2.6, 5.2, 7.0, and 10.4 mg/kg being used. PTC596 was quickly absorbed when taken orally, and the maximum plasma concentration was reached two to four hours after dosing, ranging from 0.6 to 7.0 mg/kg. Seven patients showed stable disease as the best overall response, and two received the study medication for up to 16 weeks [29]. These findings encouraged PTC596’s continued development for the treatment of solid tumors and the performing of ongoing clinical trials testing PTC596 for different types of cancers (e.g., NCT03605550, NCT03761095, and NCT03206645) [30].

Besides, there is preclinical evidence for a new treatment strategy based on targeted ablation of CSCs with PTC596 when used with cisplatin to treat patients with salivary gland adenoid cystic carcinoma [30]. The data demonstrates that this combined therapy decreases the tumorigenic potential of residual ACC tumor cells, possibly by eliminating the CSCs.

Pretreatment with quercetin decreases ionizing radiation-induced DNA double-strand breaks and cellular senescence while also reducing ROS generation, NF-κB pathway activation, and downstream proinflammatory cytokine production [28]. Moreover, quercetin decreases the release of proinflammatory cytokines, promotes wound healing, and DNA double-strand break repair by upregulating the expression of Bmi-1 [31]. Consequently, by targeting Bmi-1, quercetin can block multiple pathological processes of radiation-induced oral mucositis and could be a useful treatment option [32]. Similarly, quercetin can cause tumor cell death and alter the apoptotic pathway in cancer cells [33]. Research has demonstrated that proapoptotic protein expression can be upregulated and antiapoptotic protein expression can be diminished at a reasonable dosage of quercetin via Bmi-1 downregulation [33]. In this context, downregulation of Bmi-1 via quercetin can also reduce Bmi-1 expression and induce apoptosis in the side population in A549 cells [34].

Furthermore, a recently developed synthetic small molecule compound called QW24 exhibits strong anti-tumor activity by inhibiting Bmi-1 and stopping CSCs from self-renewing *in vivo* with no discernible toxicity, indicating that QW24 may one day be employed as a successful therapeutic agent for the treatment of colorectal cancer in clinical settings [35].

## Regulation by MiRNA

MicroRNAs, also known as miRNAs, are RNA sequences typically consisting of 21–23 nucleotides that play a role in regulating gene expression after transcription [18]. In several types of cancers, the expression of Bmi-1 is controlled by miRNAs. Research has demonstrated that miR-15 and mir-16 directly reduce the levels of Bmi-1 [36]. miR-141 has been found to trigger aging in fibroblasts by suppressing Bmi-1 [36]. Further, miR-320a targets Bmi-1 directly and prevents carcinoma of the Nasopharynx cases from expressing it [30], suggesting that different miRNAs can impact the mRNA of Bmi-1 in varying cancer situations, underscoring its significance [37].

A study suggests that tumor hypoxia has been linked to an aggressive phenotype and correlates with lower survival for cancer patients [9]. In this study, hypoxia triggered Twist1 by activating Bmi-1 expression. When either Twist1 or Bmi-1 was reduced, the EMT was reverted, and the stem cell-like properties induced by hypoxia were downregulated [9]. These findings highlight a pathway for the prognosis and treatment of hypoxic and metastatic cancers [9].

RNA interference (RNAi) is a method using molecules to inhibit target genes by breaking down specific mRNA sequences [38]. It has the advancement of easier drug delivery using cationic lipid carriers to transport short-interfering RNAs [34]. By silencing Bmi-1 through introducing siRNA and shRNA into cells via nucleofection, there is a decrease in cell proliferation and an increase in apoptosis without affecting the distribution of cell cycle phases [34]. This effect on carcinoma cells has revealed that using this approach results in inhibiting tumor formation in mice lacking functional thymus [34]. RNAi has also shown promise in overcoming chemoresistance in cell lines that overexpress Bmi-1 when treated with docetaxel, leading to the restoration of apoptosis [38].

## Current Biological Insights

Up-to-date research discernments have highlighted the role that Bmi-1 plays in mediating radio and chemoresistance. In terms of therapeutic outcomes, all these characteristics are detrimental, and they have increased the possibility of justifying Bmi-1 inhibition therapy in conjunction with traditional treatments [39]. This is especially important since HNSCC is known to be resistant to several treatments and because the disease generally has a poor prognosis at advanced stages [30].

As the carcinogenesis and progression of HNSCC are known to involve multiple genetic and epigenetic abnormalities, it is imperative to ascertain the effects of Bmi-1 targeting on normal cells. This is because it can be hypothesized that the cancer cells may be more responsive to targeted therapy than their normal counterparts. According to a study, although Bmi-1 inhibition did not significantly affect normal cells, it was selectively harmful to cancerous nasopharyngeal epithelial cells [38]. Despite not being HNSCC-specific, the results are consistent with previous research employing various cancer models and raise the prospect of a therapeutic window for Bmi-1 targeting [10,11,30,37,39].

As for Bmi-1 chemotherapeutic targeting, a subcutaneous xenograft model was used in a study to demonstrate synergistic antitumor effects when Bmi-1 RNAi and cisplatin treatment were combined [30]. The combination therapy significantly reduced the growth of the tumor and showed signs of cell cycle inhibition and apoptotic induction. Moreover, single agent PTC596 and PTC596/cisplatin combination decreased the CSC fraction in ACC. Notably, short-term combination therapy (2 weeks) with PTC596/cisplatin prevented tumor relapse for 150 days in a preclinical trial in mice [21]. According to the authors, therapeutic Bmi-1 inhibition ablates chemoresistant CSCs and rests ACC tumor relapse. These findings together indicate that Bmi-1-targeted treatments may be advantageous for ACC patients.

Nivolumab and pembrolizumab, two anti-PD-1 immunotherapies, are frequently used in conjunction with cisplatin therapy as first-line treatments for HNSCC. An *in vivo* mouse model of HNSCC has demonstrated that treatment with cisplatin and anti-PD-1 therapy enriches Bmi-1+ CSCs in tumors. Still, Bmi-1 inhibitor PTC209 therapy inhibits the initiation of these cells and the advancement of the tumor [40].

PRT4165, another Bmi-1 inhibitor, prevents the accumulation of any detectable H2A ubiquitination sites underlying DNA double-strand cuts in an osteosarcoma model. This discovery may be highly significant if combined with antiproliferative chemotherapy treatments that intensify the response to DNA damage [41]. The Bmi-1 inhibitor PTC028 has been shown to selectively inhibit the growth of cancer cells while protecting normal cells in an ovarian cancer model [42]. This may offer a distinct advantage in comparison to conventional chemotherapy medicine that does not specifically eradicate tumor cells. Although the clinical effectiveness of inhibitors of Bmi-1 for the treatment of head and neck cancers has not been extensively studied in published research, these small-molecule medications have shown promise in treating other malignancies in clinical and preclinical models, indicating that they may have novel applications in medicine.

## Genetic Treatment Protocols and Clinical Trials

Bmi-1 inhibitors are a class of pharmaceutical compounds designed to target and inhibit the Bmi-1 protein [43]. Genetic treatment protocols for Bmi-1 primarily involve strategies aimed at modulating Bmi-1 expression or function at the genetic level. However, research in genetic treatment protocols for Bmi-1 is ongoing, focusing on developing safe and effective strategies and drugs that can selectively target Bmi-1 dysregulation in cancer.

Similarly, clinical trials on humans have also examined Bmi-1 inhibitors’ potential for treatment. A multicenter phase 1 study on a patient population with advanced solid tumors found that PTC596, a second-generation Bmi-1 inhibitor, exhibited tolerable side effects that could be managed by the patients [29,41].

Ongoing clinical trials are critical to understanding the function of Bmi-1 as a target in head and neck cancers, as well as to future developments in the field of cancer therapy research. Presently, two ongoing clinical trials are using PTC-209, a novel medication that specifically inhibits Bmi-1, to treat solid tumors. Further, a phase II trial of ipilimumab in combination with nivolumab in patients with advanced nasopharyngeal carcinoma and a phase II trial of cisplatin combined with oral TS-1 in patients with advanced solid tumors with different degrees of liver dysfunction [44].

## Future Perspectives

One of the prime goals of cancer research has been understanding the nature of this disease and the different cell populations comprising the characteristic features of cancer. When the Bmi-1 protein is inactivated in these cancerous cells, denoted by the downregulation of the transcription of genes, the cell turns into a more invasive apoptotic program. Bmi-1 controls gene transcription by adding a repressive histone code. Hence, this gene is an attractive target for cancer therapy.

Reducing the cancer cell’s invasiveness and increasing its susceptibility to host defense mechanisms can be a significant drawback when exploring the inhibition of oncogenic pathways as a possible cancer therapy. In the case of HNSCC, this idea holds for Bmi-1 targeting.

Even though evidence showed that the inhibition of Bmi-1 might be a viable course for further developing anti-cancer treatment, there is much to be determined about the actual mechanisms by which the Bmi-1 gene participates in relating to the rate of tumor progression. This knowledge could be crucial in identifying patients who would be more likely to respond positively to Bmi-1 targeted therapy.

Therefore, this becomes a critical milestone in individualized cancer therapy, because when the mechanisms of the effects of Bmi-1 are identified, this can present scientists with an opportunity to localize this transcription factor as a pivotal element for identifying a certain group of patients who would benefit most from Bmi-1 targeted therapy. This could improve response rates and reduce adverse effects of the treatment.

However, it is imperative to acknowledge certain limitations of this narrative review, primarily pertaining to the accessibility of data from both ongoing and completed clinical trials. Data isn’t available in open-access statements, regardless of the patent protection of potentially novel medications or treatments. A significant improvement would be for organizations, colleges, and other establishments to announce or release information about their significant discoveries; this would improve and concentrate upcoming studies for the field’s advancement.

## Conclusion

The potential discovery of downstream effectors of Bmi-1, which could also lead to improved detection of cancers—perhaps an early application of RNAi Bmi-1 inhibition as a preventative measure in a patient identified as high risk—might hold just as much potential in developing future therapies.

## Data Availability

All data generated or analyzed during this study are included in this published article and are available from the corresponding author upon reasonable request.
